# Lonafarnib Is a Potential Inhibitor for Neovascularization

**DOI:** 10.1371/journal.pone.0122830

**Published:** 2015-04-08

**Authors:** Linlin Sun, Songbo Xie, Guoyuan Peng, Jian Wang, Yuanyuan Li, Juan Qin, Diansheng Zhong

**Affiliations:** 1 Lung Cancer Institute, Tianjin Medical University General Hospital, Tianjin, China; 2 Department of Medical Oncology, Tianjin Medical University General Hospital, Tianjin, China; 3 Department of Genetics and Cell Biology, College of Life Sciences, Nankai University, Tianjin, China; University of Kansas Medical Center, UNITED STATES

## Abstract

Atherosclerosis is a common cardiovascular disease that involves the build-up of plaque on the inner walls of the arteries. Intraplaque neovacularization has been shown to be essential in the pathogenesis of atherosclerosis. Previous studies showed that small-molecule compounds targeting farnesyl transferase have the ability to prevent atherosclerosis in apolipoprotein E-deficient mice, but the underlying mechanism remains to be elucidated. In this study, we found that lonafarnib, a specific inhibitor of farnesyl transferase, elicits inhibitory effect on vascular endothelial capillary assembly in vitro in a dose-dependent manner. In addition, we showed that lonafarnib treatment led to a dose-dependent decrease in scratch wound closure in vitro, whereas it had little effect on endothelial cell proliferation. These data indicate that lonafarnib inhibits neovascularization via directly targeting endothelial cells and disturbing their motility. Moreover, we demonstrated that pharmacological inhibition of farnesyl transferase by lonafarnib significantly impaired centrosome reorientation toward the leading edge of endothelial cells. Mechanistically, we found that the catalytic β subunit of farnesyl transferase associated with a cytoskeletal protein important for the establishment and maintenance of cell polarity. Additionally, we showed that lonafarnib remarkably inhibited the expression of the cytoskeletal protein and interrupted its interaction with farnesyl transferase. Our findings thus offer novel mechanistic insight into the protective effect of farnesyl transferase inhibitors on atherosclerosis and provide encouraging evidence for the potential use of this group of agents in inhibiting plaque neovascularization.

## Introduction

Cardiovascular diseases are the leading cause of death worldwide. Atherosclerosis is a type of cardiovascular disease that involves the build-up of plaque on the inner walls of the arteries, resulting in decreased flexibility and elasticity of these vital transports. Intraplaque neovascularization has been shown to be an essential process in atherosclerosis[1]. As one of the main characteristics of the vulnerable plaque, neovascularization has been implicated to be associated with plaque growth, leukocyte exchange and plaque instability[2]. These findings suggest that inhibition of neovascularizaton might be a therapeutic option for atherosclerosis [3,4]. However, the molecules involved in the process of neovascularizaton remain elusive.

The protein farnesyl transferase is a prenylation enzyme comprised of a common regulatory α subunit and a specific catalytic β subunit. Farnesyl transferase recognizes proteins with a COOH terminus CAAX motif and transfers a 15-carbon farnesyl group to the C-terminal cysteine[5]. Farnesylation is a posttranslational modification that is required for proteins, such as Ras, to properly localize within membrane structures[6]. Previous study showed that the small-molecule compounds targeting farnesyl transferase have the ability to prevent atherosclerosis in apolipoprotein E-deficient mice, as evidenced by reduced fatty streak lesion size, decreased smooth muscle-like cell accumulation in the neointima and ameliorated oxidative stress[7]. However, very little is known about the mechanism underlying the action of this group of compounds in atherosclerosis.

Given the important role of intraplaque neovascularization in atherosclerosis, in this study, we sought to investigate the potential effect of lonafarnib, a nonpeptide tricyclic farnesyl transferase inhibitor, on neovascularization. We found that lonafarnib elicits inhibitory effect on neovascularization via disturbing centrosome reorientation and impairing endothelial cell motility. Mechanistically, we showed that the catalytic β subunit of farnesyl transferase interacts with a cytoskeletal protein required for the regulation of microtubule dynamics[8]. Moreover, the expression of the cytoskeletal protein and its interaction with farnesyl transferase were significantly inhibited by lonafarnib. Our findings thus help to better understand the molecular mechanism underlying the protective effect of farnesyl transferase inhibitors on atherosclerosis.

## Materials and Methods

### Materials

Lonafarnib and tipifarnib were from Schering-Plough (NY, USA) and Janssen (NJ, USA), respectively. Matrigel and antibody against MAPRE1 were purchased from BD Biosciences (NY, USA). Antibodies against α-tubulin, γ-tubulin, HA, GST and HDJ-2 were obtained from Sigma–Aldrich (MO, USA). Sulforhodamine B (SRB) and 4’, 6-diamidino-2-phenylindole (DAPI) were purchased from Sigma–Aldrich (MO, US). Glutathione Sepharose 4B beads were from Promega (WI, USA). The mammalian expression plasmids for GST-tagged MAPRE1 or HA-tagged FTβ (including the various truncated forms) were constructed by insertion of each individual cDNA in frame into pEBG-GST and pCMV-HA vectors, respectively.

### Cell culture

Pooled primary human umbilical vascular endothelial cells (HUVECs) were purchased from the American Type Culture Collection (ATCC) and cultured in RPMI 1640 medium (Gibico, USA) supplemented with 10% fetal bovine serum (Gibico, USA) at 37°C in a humidified atmosphere with 5% CO2.

### Capillary assembly assay

HUVECs were seeded on 6-well plate precoated with matrigel and treated with gradient concentrations of lonafarnib. Photographs were taken 6 hours later. The degree of capillary assembly was quantified by measuring the cumulative capillary length using the Image J software (NIH).

### Cell motility assay

Confluent monolayer of HUVECs grown in 24-well plates were mechanically scratched using a 20-μl pipette tip to create the wound. Cells were then washed with phosphate-buffered saline (PBS) to remove the debris, and treated with gradient concentrations of lonafarnib in complete culture media to allow wound healing. Phase contrast images of the wound were taken 24 hours later at three random locations. The extent of wound closure was analyzed with Image J (National Institutes of Health, NIH).

### SRB assay

HUVECs grown in 96-well culture plates were treated with 10μM lonafarnib for 24 hours and fixed with 10% trichloroacetic acid and stained with 0.4% SRB dissolved in 1% acetic acid. The cells were then washed with 1% acetic acid to remove unbound dye. The protein-bound dye was extracted with 10 mM Tris base to determine the optical density at 490 nm wavelength.

### Flow Cytometry

To evaluate cell cycle progression, 2*10^6^ HUVECs were collected, washed twice with ice-cold PBS, and fixed in 70% ethanol for 24 hours. Cells were washed again with PBS and incubated with PI (20 μg/ml) and RNaseA (20 μg/ml) in PBS for 30 min in the dark. Samples were analyzed on a BD FACSCalibur flow cytometer. To examine cell death, Annexin V staining assay was performed by using the Annexin V-FITC/PI apoptosis detection kit (BD Pharmingen). Briefly, HUVECs were washed with PBS and then resuspended in the binding buffer. Cells were then incubated with fluorescein-conjugated Annexin V and PI for 15 min in the dark, suspended in the binding buffer, and then analyzed on the flow cytometer as described above.

### Immunofluorescent microscopy

HUVECs grown on glass coverslips were mechanically scratched with 20-μl pipette tip to stimulate directed cell motility. After treatment with 10 μM lonafarnib for 8 hours, cells were fixed with methanol for 5 minutes at -20°C and blocked with 2% bovine serum albumin in PBS. Cells were then incubated with primary antibodies (anti-α-tubulin and anti-γ-tubulin) and second antibodies to immunostain microtubules and centrosomes, followed by staining DNA with DAPI for 5 minutes. Coverslips were then mounted with 90% glycerol in PBS and examined by fluorescence microscopy.

### GST pull-down

HUVECs were transfected with pEBG-GST-MAPRE1 and pCMV-HA-FTβ(or the various truncated forms of MAPRE1 and FTβ). The cell lysate was incubated with glutathione Sepharose 4B beads at 4°C for 2 hours. The beads were washed extensively and boiled in the SDS loading buffer, and the proteins were detected by SDS/PAGE and Western blot.

### Western blot

Proteins were resolved by polyacrylamide gel electrophoresis and transferred onto polyvinylidene difluoride (PVDF) membranes (Millipore, Germany). The membranes were blocked in Tris-buffered saline containing 0.2% Tween 20 and 5% fat-free dry milk and incubated first with primary antibodies and then with horseradish peroxidase-conjugated secondary antibodies. Specific proteins were visualized with enhanced chemiluminescence detection reagent according to the manufacturer’s instructions (Pierce Biotechnology, USA).

### Statistical analysis

All data shown were derived from three independent experiments, and results were presented as means ± standard errors (SEM). Student’s t-test was carried out for statistical analysis.

## Results

### Lonafarnib interrupts vascular endothelial capillary assembly

**Fig 1:**
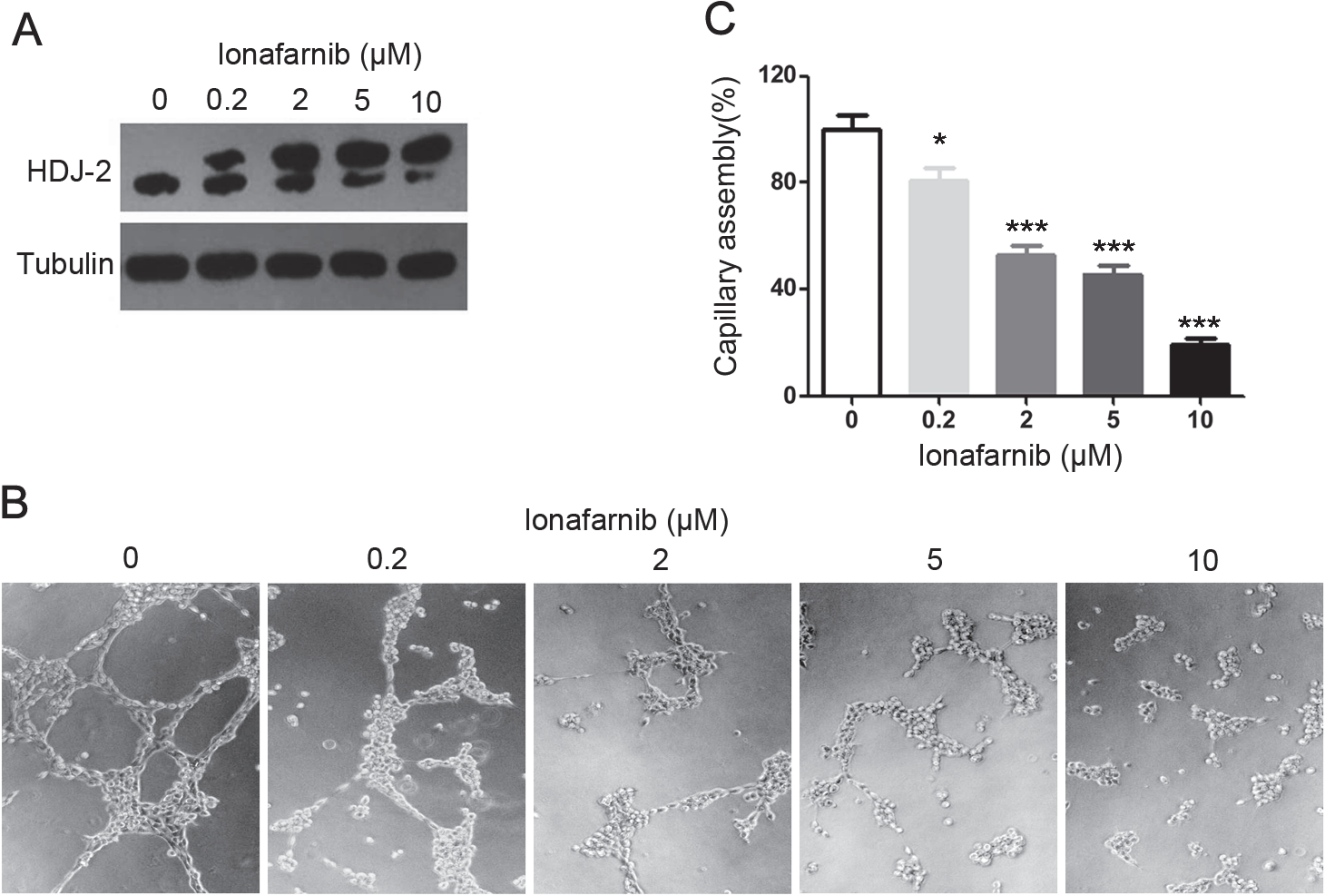
The effect of lonafarnib on vascular endothelial capillary assembly in vitro. (A) HUVECs were treated with DMSO or gradient concentrations (0.2μM -10μM) of lonafarnib for 24 hours, and HDJ-2 farnesylation was analyzed by Western blot. (B) HUVECs were plated onto matrigel and treated with DMSO or different concentrations (0.2μM -10μM) of lonafarnib. Photographs were taken 6 hours later. (C) Experiments were performed as in (B), and the cumulative capillary length was calculated. Results are means ±SEM from three independent experiments; ***P < 0.001 versus Control, *P < 0.05 versus Control.

To investigate the potential effect of lonafarnib on neovascularization in vitro, we treated HUVECs with gradient concentrations of lonafarnib and performed matrigel-based capillary assembly assay. The farnesylation of HDJ-2, one of the major substrates of farnesyl transferase, was used as a read-out for the enzyme inhibition. As shown in Fig 1A, lonafarnib inhibited HDJ-2 farnesylation in a dose-dependent manner, as demonstrated by the increase of the non-farnesylated HDJ-2 (upper band) and concomitant decrease of the farnesylated HDJ-2 (lower band). We then plated HUVECs onto matrigel and examined the effect of lonafarnib on the capillary-like structures 6 hours later. As shown in Fig 1B, lonafarnib-treated cells displayed remarkable defects in capillary assembly as compared to the control group. By measuring the cumulative capillary length, we found that lonafarnib treatment (0.2 μM -10 μM) led to a significant dose-dependent decrease in capillary assembly (Fig 1C). Thus, the data showed that lonafarnib impairs neovascularization by directly targeting the endothelial cells.

### Lonafarnib inhibits the motility of vascular endothelial cells

**Fig 2:**
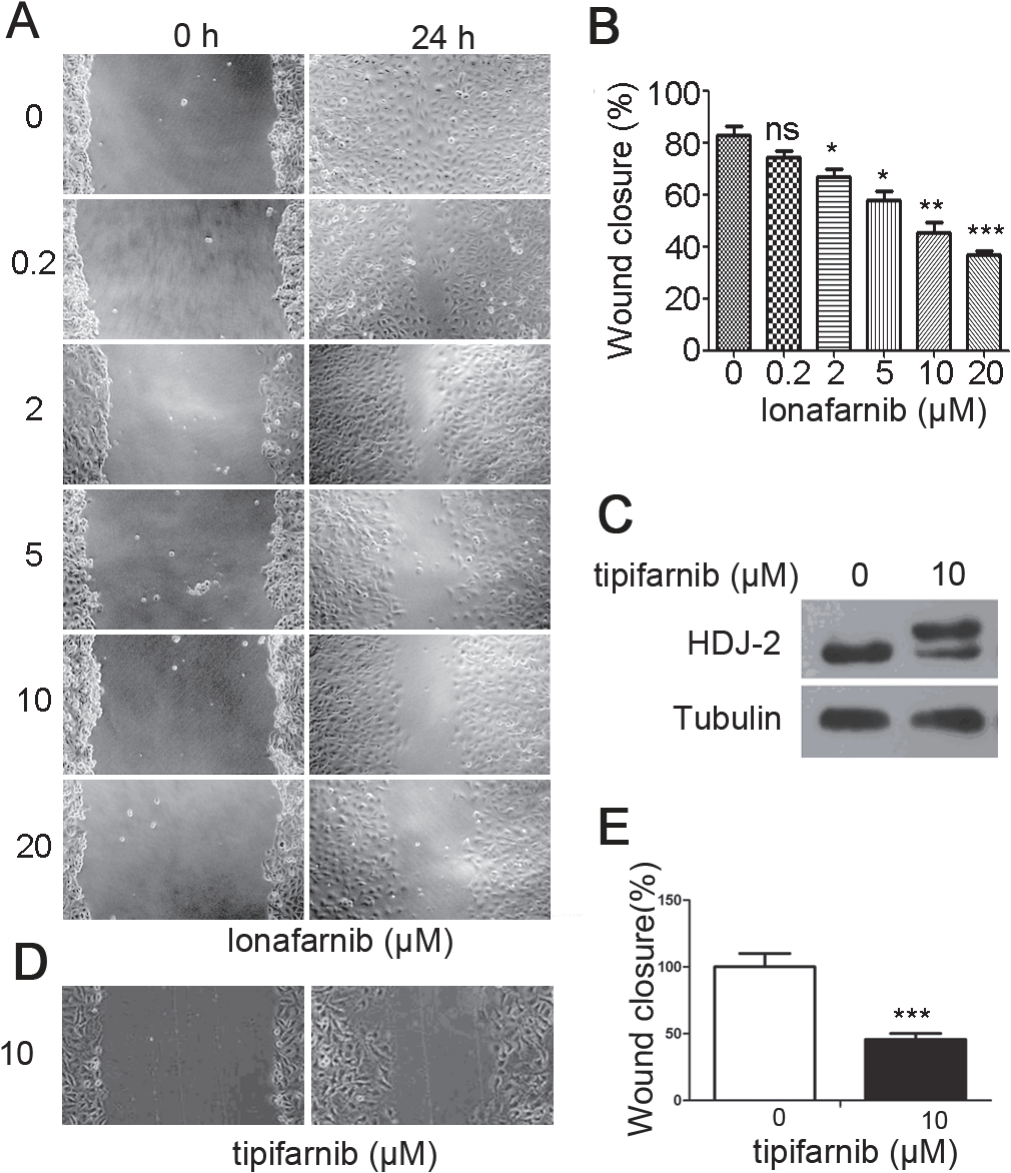
The effect of lonafarnib on the motility of vascular endothelial cells. (A) HUVECs were scratched and treated with DMSO or different concentrations (0.2μM -20μM) of lonafarnib, and wound margins were photographed 24 hours later. (B) Experiments were performed as in (A), and the extent of wound closure was quantified by measuring the wound area compared with the initial wound area. Results are means ±SEM from three independent experiments, ***P < 0.001 versus Control; **P < 0.01 versus Control; *P < 0.05 versus Control. (C) HUVECs were treated with DMSO or 10μM tipifarnib for 24 hours, and HDJ-2 farnesylation was analyzed by Western blot. (D) HUVECs were scratched and treated with DMSO or 10μM tipifarnib, and wound margins were photographed 24 hours later. (E) Experiments were performed as in (D), and the extent of wound closure was quantified by measuring the wound area compared with the initial wound area. Results are means ±SEM from three independent experiments, ***P < 0.001 versus Control.

The motility and proliferation of the endothelial cells are essential for capillary assembly[9]. We then sought to examine the effect of lonafarnib on cell motility using the standard wound healing assay. As shown in Fig 2A, in the control group, a complete wound closure was observed 24 hours after scratching in HUVECs. In contrast, there was a significant impairment in wound closure in lonafarnib-treated cells. Lonafarnib treatment (0.2 μM -20 μM) led to a dose-dependent decrease in cell motility, as demonstrated by Fig 2B. To validate our results, we treat HUVECs with tipifarnib, another specific farnesyl transferase inhibitor, which remarkably decreased HDJ-2 farnesylation (Fig 2C). Similarly, we found that tipifarnib (10 μM) was able to suppress wound closure significantly (Fig 2D and 2E), confirming the inhibitory effect of this group of agents on cell motility.

### Lonafarnib has little effect on endothelial cell proliferation

**Fig 3:**
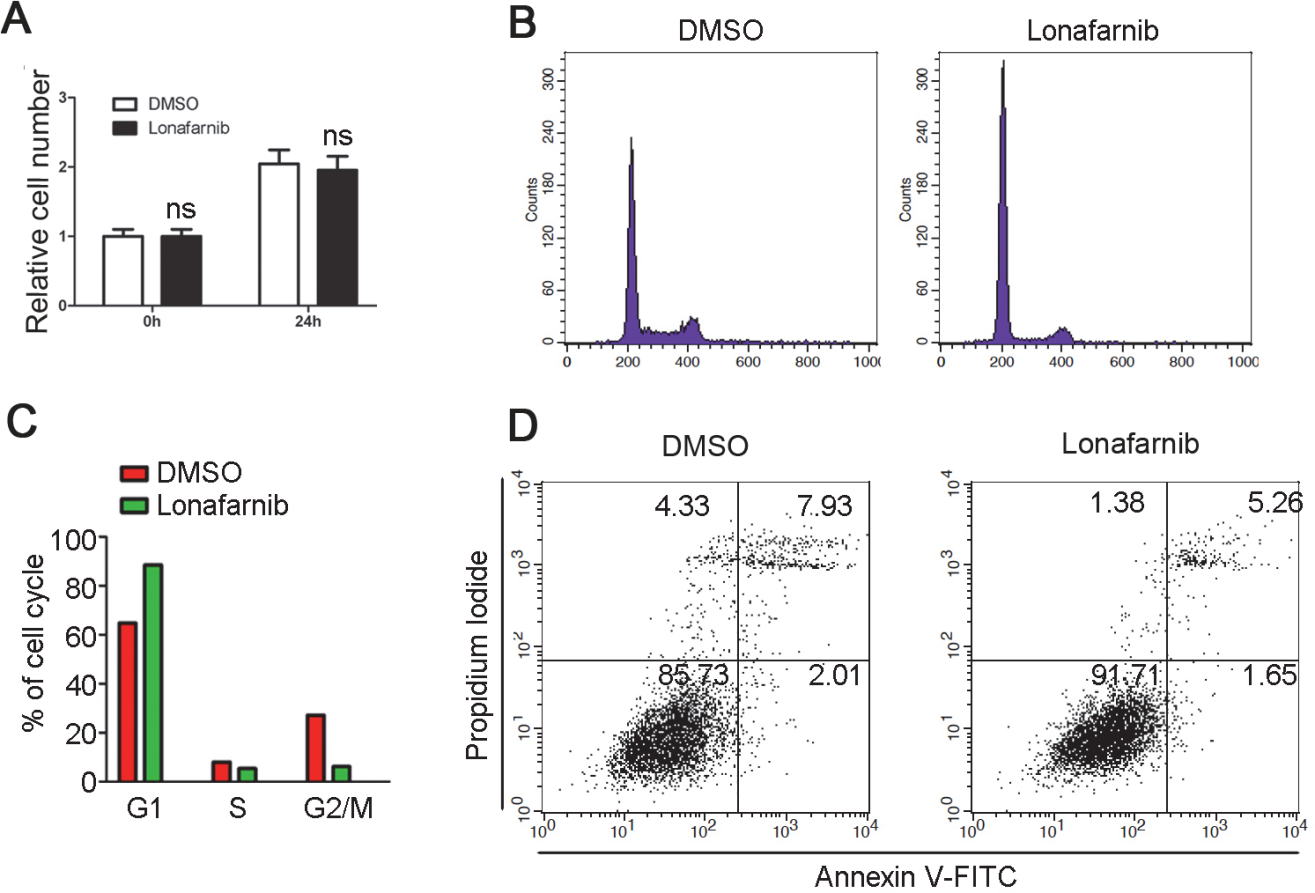
The effect of lonafarnib on the proliferation of endothelial cells. (A) HUVECs were treated with DMSO or 10μM lonafarnib for 24 hours, and SRB assay was performed to measure cell proliferation. (B)HUVECs were treated with DMSO or 10μM lonafarnib for 24 hours, stained with the DNA dye DAPI and cell cycle progression was examined by flow cytometric analysis of cellular DNA content. The amplitude of curves corresponds to the cell number. The peak on the left represents cells in G1 phase of cell cycle, while the right peak represents cells in G2/M phase. (C) Experiments were performed as in (B), and the percentage of cells in G1, S, and G2/M phases was analyzed. (D) HUVECs were treated with DMSO or 10μM lonafarnib for 24 hours, and cell death was evaluated by Annexin V/PI- staining assay.

The impaired neovascularization by lonafarnib could also result from its effect on cell proliferation, in addition to the inhibition of cell motility. To investigate the possibility, HUVECs were treated with DMSO or 10 μM lonafarnib for 24 hours and sulforhodamine B (SRB) assay was performed to measure cell proliferation. As shown in Fig 3A, lonafarnib had little effect on endothelial cell proliferation. By flow cytometric analysis of cellular DNA content, we evaluated the effect of lonafarnib on cell cycle. As shown in Fig 3B and 3C, lonafarnib treatment (10 μM) for 24 hours led to a slight increase in the number of G1 phase cells, suggesting that it might halt cell cycle progression and induced growth arrest upon prolonged treatment (see S1 Fig). We also examined the effect of lonafarnib on cell death with Annexin V/PI-staining assay. As shown in Fig 3D, we found that lonafarnib did not significantly affect cell death. Collectively, these results demonstrated that lonafarnib inhibits neovascularization via its effect on the motility, but not on the proliferation of endothelial cells.

### Lonafarnib disturbs centrosome reorientation in vascular endothelial cells

**Fig 4:**
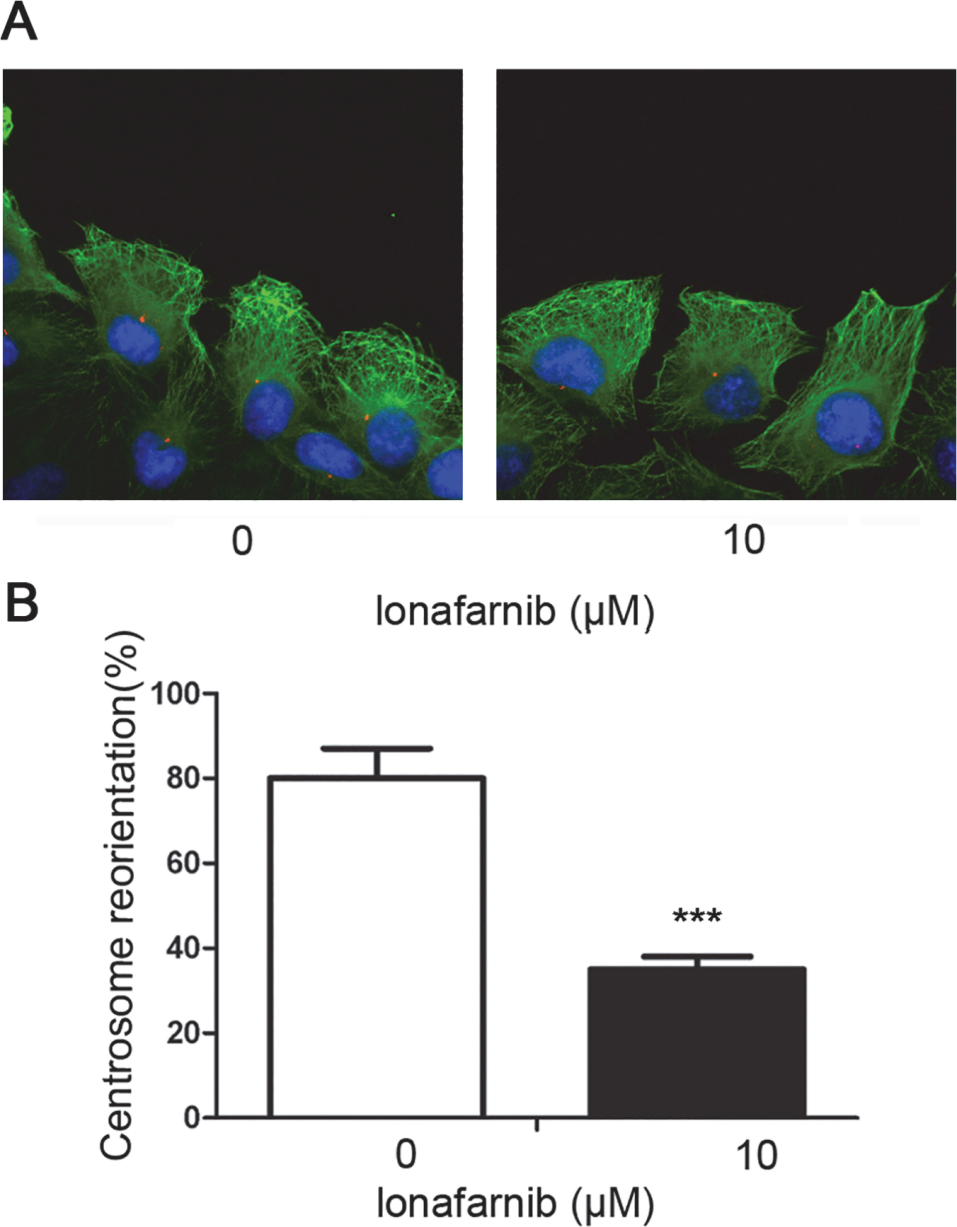
The effect of lonafarnib on centrosome reorientation in vascular endothelial cells. (A) HUVECs were scratched and treated with DMSO or 10 μM lonafarnib for 8 hours. Cells were then fixed and stained with anti-α-tubulin antibody, anti-γ-tubulin antibody and DAPI to visualize microtubules(green), centrosomes (red) and nuclei (blue), respectively. (B) Experiments were performed as in (A), and the percentage of polarized cells with proper centrosome reorientation at the wound margin was quantified. Results are means ±SEM from three independent experiments, ***P < 0.001 versus Control.

To gain more mechanistic insight into the inhibition of neovascularization by lonafarnib, we evaluated the effect of lonafarnib on the reorientation of the centrosome towards the leading edge of cells, which is a key step for endothelial cell motility[10]. HUVECs were scratched and treated with 10 μM lonafarnib for 8 hours. Cells were then fixed and immunostained to visualize microtubules, centrosomes and nuclei., As shown in the representative image in Fig 4A and quantified in Fig 4B, in the control group, cells at the wound margin exhibited a typical polarized morphology with centrosomes localized between the nuclei and the leading edge. In contrast, lonafarnib-treated cells displayed significant defects in the position of centrosomes, which randomly localized and failed to properly orient themselves to the direction of motility. Thus, the data showed that lonafarnib significantly disturbs the reorientation of centrosome in the motile vascular endothelial cells.

### FTβinteracts with MAPRE1 in vascular endothelial cells

**Fig 5:**
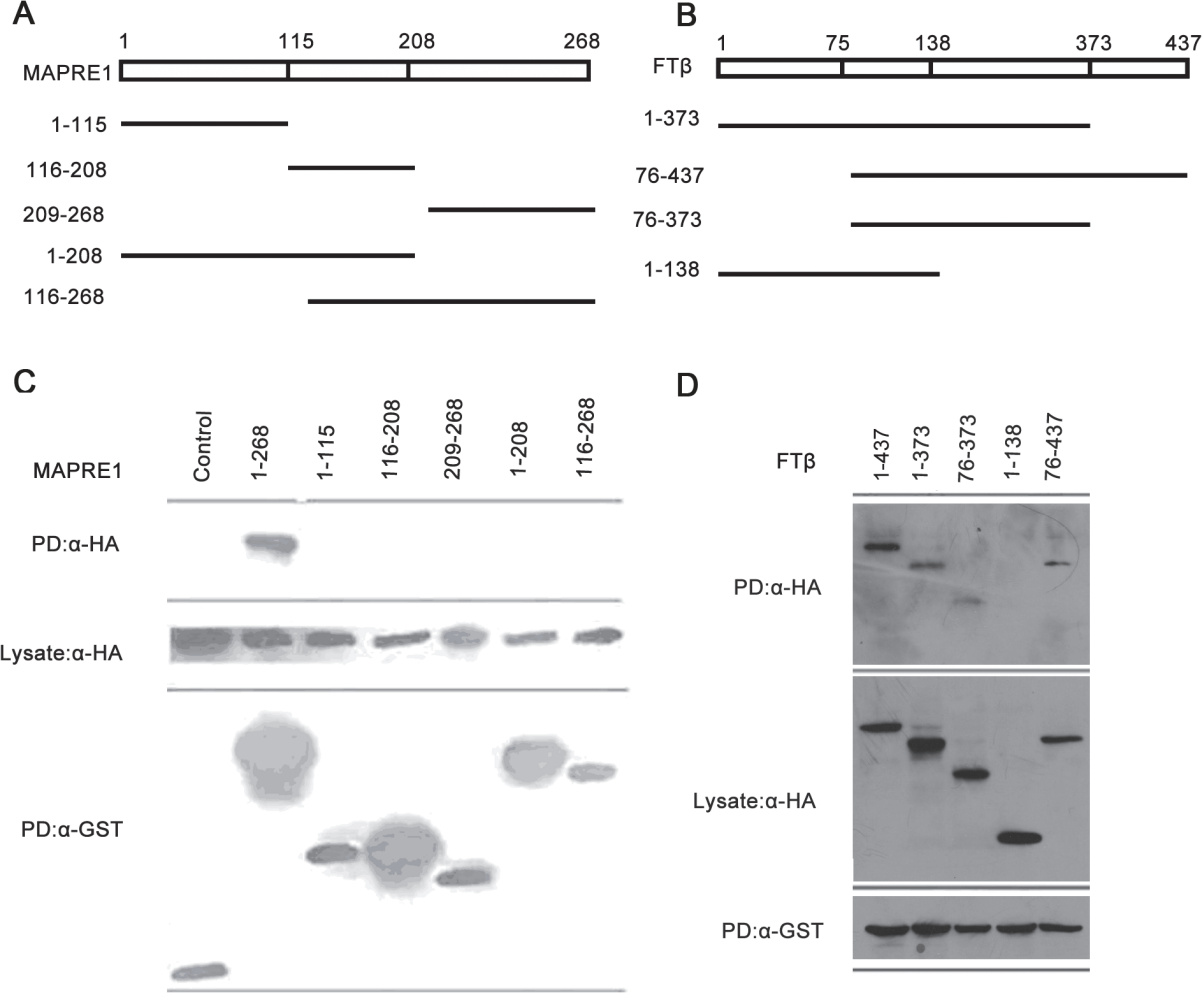
Characterization of the interaction between MAPRE1 and FTβ. (A) Schematic representations of full length (FL) and truncated forms of MAPRE1 were shown. (B) HUVECs were transfected with pCMV-HA-FTβand plasmids that express various truncated forms of MAPRE1 tagged with GST. GST pull-down and Western blot were then performed to characterize the FTβbinding region on MAPRE1. The expressions of the MAPRE1 variants were monitored and comparable amounts of cell lysates were loaded in the pull-down assay (lower panel). (C) Schematic representations of full length (FL) and truncated forms of FTβwere shown. (D) HUVECs were transfected with GST-MAPRE1 and plasmids that express various truncated forms of FTβtagged with HA. GST pull-down (PD) and Western blot were then performed to examine their interaction and characterize the MAPRE1 interacting region on FTβ. The expressions of the FTβvariants were monitored and comparable amounts of cell lysates were loaded in the pull-down assay (middle panel).

The findings that pharmacological inhibition of farnesyl transferase by lonafarnib impaired the position of centrosome suggest that the protein might function in the process of centrosome reorientation. In an effort to elucidate the underlying molecular mechanism, we found that the catalytic β subunit of farnesyl transferase appeared to associate with a cytoskeletal protein named microtubule-associated protein RP/EB family member 1(MAPRE1), a key regulator of cell polarization[8]. To confirm our observation, a series of truncated forms of MAPRE1 tagged with GST were constructed, and the representative truncations were depicted in Fig 5A. HUVECs were then transfected with these plasmids together with the HA-FTβ-expressing plasmid, and cell lysates were analyzed by GST pull-down assay. As shown in Fig 5B, GST-MAPRE1 was able to pull down HA-FTβ, validating the association of farnesyl transferaseβ with MAPRE1. In addition, the full length of MAPRE1 (1–268) was required for the interaction with FTβ, and other truncations of MAPRE1 (amino acids 1–115, 116–208, 209–268, 1–208, 116–268) abrogated the binding with FTβ. Similarly, by constructing various truncated forms of FTβ(Fig 5C), we sought to identify the MAPRE1 binding region in FTβ. GST pull-down assay revealed that amino acids 1–373, 76–437 and 76–373 of FTβwere able to interact with GST-MAPRE1 but that amino acids 1–138 abrogated the association with MAPRE1 (Fig 5D), indicating that amino acids 139–373 of FTβwere essential for their interaction. These data thus identified MAPRE1 as a binding partner of farnesyl transferase in vascular endothelial cells.

### Lonafarnib suppresses MAPRE1 expression and interrupts its interaction with FTβ

**Fig 6:**
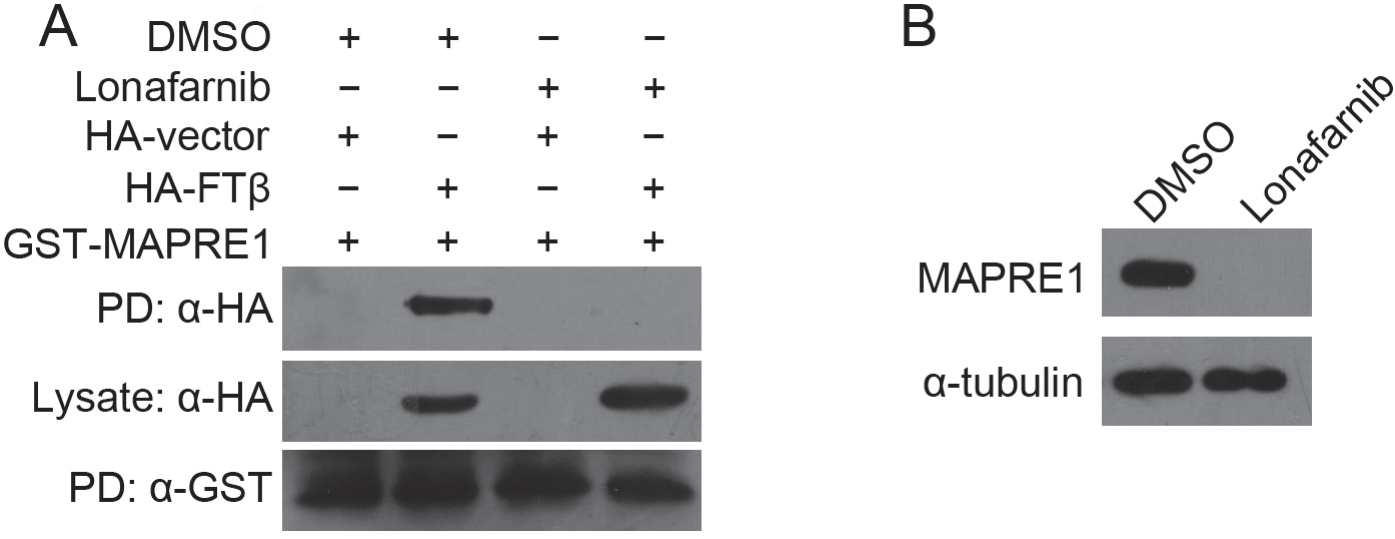
Lonafarnib suppresses MAPRE1 expression and interrupts its interaction with FTβ. (A) After co-transfection of GST-MAPRE1 and HA-FTβ, HUVECs were treated with DMSO or 10 μM lonafarnib for 24 hours. GST pull-down (PD) and Western blot were then performed to examine their interaction. The expressions of the HA-FTβwere monitored and comparable amounts of cell lysates were loaded in the pull-down assay (middle panel). (B) HUVECs were treated with DMSO or 10 μM lonafarnib for 24 hours, and endogenous MAPRE1expession was analyzed by Western blot.

Next, we sought to investigate whether lonafarnib affects the interaction of FTβwith MAPRE1. HUVECs were co-transfected with GST-MAPRE1 and HA-FTβ and then treated with DMSO or 10 μM lonafarnib for 24 hours. By GST pull-down assay, we found that lonafarnib significantly decreased the association between GST-MAPRE1 and HA-FTβ, compared with the control group (Fig 6A). Moreover, we also evaluated the effect of lonafarnib on the endogenous expression of MAPRE1. As shown in Fig 6B, we found that 10 μM lonafarnib treatment for 24 hours remarkably inhibited the expression of MAPRE1. Thus, these data showed that lonafarnib potently suppressed the expression of MAPRE1 and its association with FTβ.

## Discussion

Farnesyl transferase inhibitors have been originally developed to pharmacologically block the transformation by Ras [6]. Although much has been learned on their anti-tumor activity [11], the effect of these agents on cardiovascular diseases has received much less attention. Intriguingly, it has been reported that farnesyl transferase inhibitors demonstrate the ability to prevent atherosclerosis in apolipoprotein E-deficient mice [7], but the biology behind its action remains an open question. In the present study, we showed that lonafarnib, one of the first farnesyl transferase inhibitors to undergo clinical trials [12], directly targets vascular endothelial cells and inhibits neovascularization. Given the importance of plaque neovascularization in the pathogenesis of atherosclerosis, we proposed that, by inhibiting endothelial cell-induced intraplaque neovascularization, lonafarnib might affect plaque growth, leukocyte exchange or plaque vulnerability, and thus exert its preventive effect on atherosclerosis. These questions will be addressed in future with in vivo model of atherosclerosis. In addition, our data seem to be in accord with a previous study reporting that A-170634, a specific farnesyl transferase inhibitor, impaired VEGF-stimulated angiogenesis in a rat corneal angiogenesis model [13]. Our study thus significantly increases our understanding of the protective effects of farnesyl transferase inhibitors in the context of cardiovascular diseases and provides encouraging evidence for the potential use of these agents in the treatment of atherosclerosis[14].

The process of neovascularization involves a series of key events, In response to angiogenic signals, vascular endothelial cells migrate and proliferate to form provisional tubes. Thus, the motility of endothelial cells is required for the vascular sprouting of introplaque neovascularization[15]. By wound healing assays, we clearly demonstrate that lonafarnib inhibits neovascularization through its actions on endothelial cell motility, not on cell proliferation. In good agreement with our study, L-744,832, another specific inhibitor of farnesyl transferase has been shown to block the directional chemotaxis of endothelial cells toward VEGF [16]. Centrosome reorientation, a characteristic polarized morphology, is an essential step for cell motility. In this study, we have shown by immunofluorescence microscopy that lonafarnib impairs the reorientation of centrosome toward the leading edge of cells. Collectively, our findings indicate that lonafarnib inhibits neovascularization via interrupting centrosome reorientation and decreasing endothelial cell motility.

Importantly, our study provides the mechanistic insight into why pharmacological inhibition of farnesyl transferase by lonafarnib impairs the centrosome reorientation. We showed that catalytic β subunit of farnesyl transferase interacts with MAPRE1, a microtubule associated protein (MAP) critical for microtubule dynamics and cell polarity. Specifically, the amino acids 138–373 of farnesyl transferase and the full length of MAPRE1 are required for their interaction. The findings thus suggest the potential role of MAPRE1 in mediating the function of farnesyl transferase in the process of centrosome reorientation. Intriguingly, the active form of the enzyme (bothαand -βsubunits) has been previously shown to bind with microtubules directly[17]. Thus, whether farnesyl transferase interacts with MAPRE1 directly, or indirectly with microtubules providing the dynamic scaffold for their interaction, remains unclear. Future studies are needed to address these questions. Certainly, since farnesyl transferase associates with a great number of proteins in cells, it might function in centrosome reorientation through alternative mechanisms in addition to the interaction with MAPRE1.

Farnesyl transferase is known to function primarily through the farnesylation of its substrate proteins, such as CENP-A, myosin II regulatory light chain and INCENP[5]. Intriguingly, MAPRE1 does not possess a CAAX farnesylation motif, so it does not belong to the family of “classic” target proteins of farnesyl transferase and is not a direct substrate of the enzyme. This raises questions about whether and how farnesyl transferase regulates MAPRE1 function. It is possible that additional proteins, potentially farnesylated, are present in the complex and could mediate the proper localization of MAPRE1 on the microtubule tips, or, reciprocally, as with MAPRE1, farnesyl transferase might localizes to the plus end of microtubules and farnesylate its substrates for translocating into cell membrane. It will be interesting to investigate these questions in the future and explore how farnesyl transferase coordinates with MAPRE1 to regulate centrosome reorientation. Additionally, we found that lonafarnib decreased the expression of MAPRE1 and its interaction with farnesyl transferase, thus providing the potential molecular mechanism by which lonafarnib inhibits centrosome reoriendation and endothelial cell motility. Furthermore, given the essential role of MAPRE1 in a wide spectrum of cellular processes, such as search and capture of chromosomes during mitosis[18], it is reasonably conceivable that lonafarnib might affect these processes via suppressing MAPRE1 expression or its interaction with farnesyl transferase.

In conclusion, our study showed that lonafarnib, a specific inhibitor of farnesyl transferase, inhibits neovascularization via directly targeting endothelial cells. Based on our results, we proposed that, by decreasing the MAPRE1 expression and its interaction with farnesyl transferase, lonafarnib interrupts centrosome reorientation and thus slows endothelial cell motility. Collectively, our findings offer novel mechanistic insight into the protective effect of farnesyl transferase inhibitors on atherosclerosis and provide encouraging evidence for the potential use of this group of agents in inhibiting plaque neovascularization.

## Supporting Information

S1 Fig Prolonged treatment with lonafarnib inhibits endothelial cell proliferation. HUVECs were treated with DMSO or 10μM lonafarnib for 24 or 48 hours, and SRB assay was performed to measure cell proliferation.
(TIF)
