# Supplementary figures and images for: Lonafarnib Is a Potential Inhibitor for Neovascularization
Source: PLoS One. 2015 Apr 8;10(4):e0122830. doi: 10.1371/journal.pone.0122830 (PMC4390146; doi:10.1371/journal.pone.0122830)

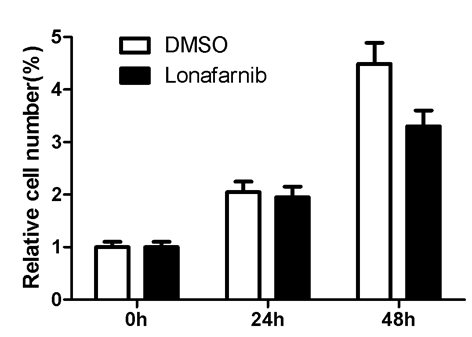

Supplement: S1 Fig — HUVECs were treated with DMSO or 10μM lonafarnib for 24 or 48 hours, and SRB assay was performed to measure cell proliferation. (TIF) [file pone.0122830.s001.tif]
